# Tanshinone IIA and Cryptotanshinone Counteract Inflammation by Regulating Gene and miRNA Expression in Human SGBS Adipocytes

**DOI:** 10.3390/biom13071029

**Published:** 2023-06-23

**Authors:** Sara Carpi, Stefano Quarta, Stefano Doccini, Anella Saviano, Noemi Marigliano, Beatrice Polini, Marika Massaro, Maria Annunziata Carluccio, Nadia Calabriso, Martin Wabitsch, Filippo Maria Santorelli, Marco Cecchini, Francesco Maione, Paola Nieri, Egeria Scoditti

**Affiliations:** 1 Science of Health Department, Magna Græcia University, 88100 Catanzaro, Italy; sara.carpi@unicz.it; 2 NEST, Istituto Nanoscienze-CNR and Scuola Normale Superiore, 56100 Pisa, Italy; marco.cecchini@nano.cnr.it; 3 Department of Pharmacy, University of Pisa, 56100 Pisa, Italy; beatrice.polini@farm.unipi.it (B.P.); paola.nieri@unipi.it (P.N.); 4 Department of Biological and Environmental Sciences and Technologies (DISTEBA), University of Salento, 73100 Lecce, Italy; stefano.quarta3@unisalento.it; 5 IRCCS Fondazione Stella Maris, Calambrone, 56128 Pisa, Italy; stefano.doccini@fsm.unipi.it (S.D.); filippo.santorelli@fsm.unipi.it (F.M.S.); 6 ImmunoPharmaLab, Department of Pharmacy, School of Medicine and Surgery, University of Naples Federico II, 80131 Naples, Italy; anella.saviano@unina.it (A.S.); noemi.marigliano@outlook.com (N.M.); francesco.maione@unina.it (F.M.); 7 Department of Pathology, University of Pisa, 56100 Pisa, Italy; 8 National Research Council (CNR), Institute of Clinical Physiology (IFC), 73100 Lecce, Italy; marika.massaro@ifc.cnr.it (M.M.); maria.carluccio@ifc.cnr.it (M.A.C.); nadia.calabriso@ifc.cnr.it (N.C.); 9 Division of Pediatric Endocrinology, Diabetes and Obesity, Department of Pediatrics and Adolescent Medicine, University of Ulm, 89075 Ulm, Germany; martin.wabitsch@uniklinik-ulm.de

**Keywords:** adipocyte, chemokine, cryptotanshinone, cytokine, Danshen, gene expression, inflammation, miRNA, obesity, *Salvia milthorrhiza* bunge, tanshinone-IIA

## Abstract

Inflammation of the adipose tissue contributes to the onset and progression of several chronic obesity-related diseases. The two most important lipophilic diterpenoid compounds found in the root of *Salvia milthorrhiza* Bunge (also called Danshen), tanshinone IIA (TIIA) and cryptotanshinone (CRY), have many favorable pharmacological effects. However, their roles in obesity-associated adipocyte inflammation and related sub-networks have not been fully elucidated. In the present study, we investigated the gene, miRNAs and protein expression profile of prototypical obesity-associated dysfunction markers in inflamed human adipocytes treated with TIIA and CRY. The results showed that TIIA and CRY prevented tumor necrosis factor (TNF)-α induced inflammatory response in adipocytes, by counter-regulating the pattern of secreted cytokines/chemokines associated with adipocyte inflammation (CCL2/MCP-1, CXCL10/IP-10, CCL5/RANTES, CXCL1/GRO-α, IL-6, IL-8, MIF and PAI-1/Serpin E1) via the modulation of gene expression (as demonstrated for CCL2/MCP-1, CXCL10/IP-10, CCL5/RANTES, CXCL1/GRO-α, and IL-8), as well as related miRNA expression (miR-126-3p, miR-223-3p, miR-124-3p, miR-155-5p, and miR-132-3p), and by attenuating monocyte recruitment. This is the first demonstration of a beneficial effect by TIIA and CRY on adipocyte dysfunction associated with obesity development and complications, offering a new outlook for the prevention and/or treatment of metabolic diseases.

## 1. Introduction

Fat accumulation in the visceral adipose depot and consequent obesity are important risk factors for type 2 diabetes, non-alcoholic fatty liver disease (NAFLD), dyslipidemia, hypertension, cardiovascular disease, and cancer [1,2,3]. Far from being simply the result of an energy imbalance between calorie intake and expenditure, obesity involves a host of metabolic alterations, including mitochondrial dysfunction, oxidative stress, immune response, and chronic low-grade inflammation, which are significant correlates and possible determinants of the increased cardiometabolic risk associated with visceral obesity [1]. Under prolonged positive energy balance conditions, chronic fat accumulation in white adipose tissue leads to hypertrophic expansion of adipocytes, cell stress, and eventually death, along with impaired adipogenesis, altered tissue perfusion, and fibrosis [4]. As a consequence, adipocytes become metabolically dysfunctional, acquiring a pro-lipolytic and insulin-resistant phenotype, with consequent free fatty acid (FFA) spillover into the circulation and ectopic lipid accumulation, such as in the liver, skeletal muscle, and heart, which promotes local inflammation, insulin resistance, and organ damage [4]. Furthermore, adipose tissue expansion is associated with a shift of the transcriptional program of hypertrophic adipocytes toward a pro-inflammatory, diabetogenic, and proatherogenic pattern of adipokines, i.e., adipose tissue secretes bioactive factors [4,5,6]. In fact, the secretion of anti-inflammatory and insulin-sensitizing adipokines, such as adiponectin, is downregulated, while the secreted levels of pro-inflammatory mediators, such as interleukin (IL)-6, IL-8, monocyte chemoattractant protein (MCP)-1, chemokine (C-C motif) ligand 5 (CCL-5) and tumor necrosis factor (TNF)-α, increase [7]. In particular, chemokines production by adipocytes promotes the recruitment and infiltration of immune cells, mostly macrophages but also neutrophils, T cells, and B2 cells, in the adipose tissue [8]. Here, the dysmetabolic microenvironment of obese adipose tissue leads to pro-inflammatory activation of recruited immune cells; indeed, recruited adipose tissue macrophages in obesity differentiate into a M1 proinflammatory state, thus causing an imbalance between M1 and anti-inflammatory M2 macrophages [9,10]. Increased production of inflammatory chemokines and cytokines from these cells amplifies the inflamed status of the adipose tissue in a vicious cycle and inhibits the insulin signaling cascade [9,10]. Several studies have shown that decreased adipose tissue macrophage recruitment is associated with reduced adipose tissue inflammation and insulin resistance in obesity [11,12,13]. The altered pattern of adipose tissue-released cytokines/adipokines, lipids, and microRNA (miRNA)-containing exosomal vesicles, which travel through the circulation to influence distant organs, contributes to establishing a state of systemic chronic low-grade inflammation that is a pathogenic link between obesity and cardiometabolic complications [7].

Inflammation of adipocytes and immune cell activation/interactions in adipose tissue offer an attractive therapeutic target for treating obesity-related metabolic and cardiovascular conditions [14]. Neutralizing antibodies against cytokines, including TNF-α and IL-1β, have been shown to provide anti-diabetic effects in clinical studies [14]. Moreover, genetic deletion [15,16] or pharmacological antagonism of inflammatory pathways, for example, by using a high dose of salicylates [17] or chemokine antagonists [18], have been shown to alleviate obesity-induced inflammation and insulin resistance.

Currently, there are several pharmacological strategies, surgical approaches, and lifestyle regimens (e.g., diet and exercise) for the treatment of obesity, although they provide only limited long-term success. Research on natural compounds in different plants provides a growing arsenal of potential pharmaceutical agents using ethnopharmacological information. Indeed, a large percentage of marketed therapeutics (about 40%) rely on non-modified or semi-synthetic natural compounds with biological activity [19]. Consequently, in the past few decades, researchers have paid much attention to the active compounds present in medicinal and/or edible plants and their effects on human diseases, including obesity management [20,21].

**Figure 1:**
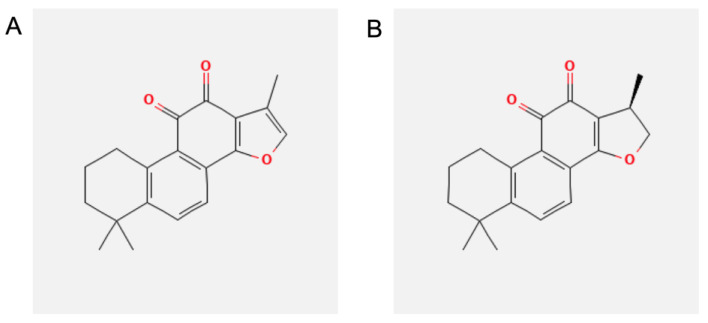
Chemical structure of (**A**) tanshinone IIA (TIIA) and (**B**) cryptotanshinone (CRY).

In this context, a novel potential natural compound-based approach could be found in the root of *Salvia milthorrhiza* Bunge (also referred to as Danshen), a perennial herb belonging to the *Labiatae* family [22]. The dry root and/or rhizome of *Salvia milthorrhiza* Bunge is officially listed in the Chines Pharmacopoeia [23] and has been one of the main commercial medicinal herbs in China for over a thousand years. The main uses are to treat inflammatory [24,25], cardiovascular [26,27], cerebrovascular [28,29] disorders and other pathological conditions [30,31,32]. Moreover, extracts of *S. milthorrhiza* roots named “Compound Danshen Dripping pill” are investigated in six different clinical trials in China and the United States in conditions such as angina pectoris, coronary heart disease, diabetes and diabetic retinopathy [33]. The favorable pharmacological effects are mainly attributed to lipophilic diterpenoid compounds, such as tanshinone IIA (TIIA) and cryptotanshinone (CRY) (Figure 1) [34].

Indeed, TIIA has been shown to reduce inflammatory cytokine production induced by TNF-α in rheumatoid arthritis fibroblast-like synoviocytes and to ameliorate arthritis severity in adjuvant-induced arthritis mice models [35]. Moreover, TIIA inhibited key inflammatory factors, such as IL-1β, in lipopolysaccharide (LPS)-stimulated RAW264.7 macrophages [36,37] and has been shown to have immune-regulating and anti-inflammatory roles in murine models [38,39]. Similarly, CRY has been shown to suppress the production of pro-inflammatory cytokines both in in vitro and in vivo models [40] and to inhibit the nuclear factor-κB (NF-κB) pathway, a pro-inflammatory transcription factor, in immune cells, including THP-1 monocytes and RAW264.7 macrophages [41,42].

Despite the potential TIIA and CRY as anti-inflammatory agents, no experimental study has been conducted to determine their effects on human adipocyte models of inflammation and obesity. Against this background, we hypothesized that TIIA and CRY might modulate the obesity-associated adipocyte inflammatory response. We, therefore, evaluated the effects of TIIA and CRY on the expression of genes, proteins and miRNAs involved in TNF-α-induced inflammatory phenotype of human adipocytes and explored underlying molecular mechanisms.

We here demonstrate that TIIA and CRY can improve adipocyte dysfunction by beneficially regulating the expression of mRNAs, proteins and miRNAs in inflamed adipocytes and attenuating monocyte recruitment.

## 2. Materials and Methods

### 2.1. Materials

TIIA and CRY (≥98% purity) were obtained from Sigma Aldrich (now under Merck, Darmstadt, Germany) and dissolved in dimethyl sulfoxide (DMSO). All other chemicals were obtained from Sigma Aldrich, unless otherwise indicated. The proteome profiler human cytokine array kit (Catalog #ARY005B) was purchased from R&D System (Milan, Italy). Unless otherwise stated, all the other reagents were from BioCell (Milan, Italy).

### 2.2. Cell Cultures and Treatments

We used human Simpson–Golabi–Behmel syndrome (SGBS) preadipocytes, a physiologically relevant cell model system resembling human adipose tissue [43]. These were a generous gift from our co-author, Prof. Martin Wabitsch (Division of Pediatric Endocrinology, Diabetes, and Obesity, Department of Pediatrics and Adolescent Medicine, University of Ulm, Ulm, Germany), and were cultured and differentiated into mature adipocytes as previously described [44]. On day 14 postdifferentiation, >90% of these cells undergo complete differentiation into mature adipocytes, as assessed using Oil Red-O lipid staining and the expression of adipocyte-specific mRNAs, such as lipoprotein lipase, adipocyte fatty acid binding protein (FABP4), peroxisome proliferator-activated receptor (PPAR), and the glucose transporter GLUT-4.

Adipose tissue inflammation was mimicked by exposing SGBS adipocytes to the pro-inflammatory cytokine TNF-α at 10 ng/mL for 18 h. Though this concentration is higher compared with those found in the circulation in obesity [45], potentially high concentrations could be found in the interstitial fluid of adipose tissue in obese subjects [46,47,48,49], thus making (patho)physiologically relevant the 10 ng/mL TNF-α concentration as used in the present study as well as in other in vitro studies [50,51,52,53]. Unstimulated controls were adipocytes incubated without TNF-α. For TIIA and CRY treatment, SGBS cells were incubated with 10 µM TIIA or CRY for 1 h before stimulation with TNF-α.

Human monocytoid THP-1 cells were obtained from the American Tissue Culture Collection (Rockville, MD, USA) and maintained in RPMI 1640 medium supplemented with 2 mmol/L glutamine, 100 mg/mL streptomycin, 100 IU/mL penicillin, and 10% FBS in a 5% CO_2_ humidified atmosphere at 37 °C.

### 2.3. Cell Viability

Cell viability was determined by the 3(4,5dimethylthiazol2yl) 2,5diphenyltetrazolium bromide (MTT) assay, as previously described [44].

### 2.4. RNA Isolation and Real-Time Quantitative Polymerase Chain Reaction

Total RNA was isolated using the TRIzol reagent (Invitrogen, Carlsbad, CA, USA) according to the manufacturer’s protocol. For real-time quantitative polymerase chain reaction (qPCR), total RNA (2 µg) was converted into first-strand cDNA by using the High Capacity cDNA Reverse Transcription Kit (Applied Biosystems, Monza, Italy). The qPCR was performed in a CFX Connect Real-Time PCR Detection System (Bio-Rad Laboratories, Milan, Italy) by using primer sequences (Table 1) for the indicated adipokines. All reactions were performed in triplicate, and the amount of mRNA was calculated by the comparative critical threshold (CT) method. To account for possible variations related to cDNA input or the presence of PCR inhibitors, the endogenous reference gene ribosomal 18S was simultaneously quantified for each sample, and the data were normalized accordingly. Results are expressed as a fold increase relative to the unstimulated control (=1).

### 2.5. Cytokines and Chemokines Protein Array

According to the manufacturer’s instructions, equal volumes (1.5 mL) of the pulled cell supernatants from all experimental conditions were then incubated with the pre-coated proteome profiler array membranes. Dot plots were detected using the enhanced chemiluminescence detection kit and Image Quant 400 GE Healthcare software (GE Healthcare, Italy) and successively quantified using GS 800 imaging densitometer software (Biorad, Italy) [54,55].

### 2.6. Evaluation of miRNAs Expression in Adipocytes

Total microRNAs (miRNAs) were extracted and purified from frozen adipocytes by using miRNeasy Mini Kit (Qiagen, Germany). Reverse transcription of the extracted miRNAs was performed by the miScript Reverse Transcription Kit (Qiagen, Germany). cDNA was diluted 1:3 in RNase-free water and then qPCRs were performed in triplicate using the miScript SYBR-Green PCR kit (Qiagen, Germany) on the MiniOpticon CFX 48 real-time PCR Detection System (Bio-Rad, Hercules, CA, USA) [56,57]. MiScript Primer Assays specific for hsa-miR-126-3p, hsa-miR-223-3p, hsa-miR-124-3p, hsa-miR-155-5p and hsa-miR-132-3p and hsa-RNU6 were obtained from Qiagen, and the probe sequences are proprietary. miRNAs expression was calculated using the Ct method and normalized to the expression of housekeeping RNU6. Mature miRNA sequences and miRBase Accession are reported in Table 2.

### 2.7. miRNA Target Prediction and Pathway Analysis

The bioinformatic survey was carried out using the Ingenuity Pathway Analysis software platform (IPA Winter Release–December 2022; QIAGEN, Hilden, Germany) [58] to recognize meaningful biological processes, disease annotations, and molecular interactions. Interaction networks were generated by selecting relations experimentally observed (maximum confidence) and functional annotations based on the number of interactions with the analyzed miRNA or mRNA targets (5) and their significance. P-value, which was ascertained by right-tailed Fisher’s Exact Test following Benjamini and Hochberg (B-H) correction, indicating the robustness of correlations. Moreover, the IPA algorithm estimated the predicted activation or inhibition of a given connecting node or biological function, simulating the directional consequences associated with the modulated miRNAs or mRNAs.

### 2.8. Leukocyte-Adipocyte Adhesion Assay

SGBS cells were treated with TIIA or CRY at the indicated concentrations for 1 h and then stimulated with 10 ng/mL TNF-α for an additional 18 h. THP-1 cells (10^6^ cells/mL) were fluorescently labeled by incubation with calcein-AM (5 ng/mL) for 30 min and then washed twice in RPMI medium. Suspended THP-1 cells were then added to the SGBS monolayers. Nonadherent cells were then removed, and monolayers were fixed with 1% paraformaldehyde. Images of SGBS and adherent calcein-labeled THP-1 cells were visualized and captured with a stereomicroscope (Nikon, Minato, Tokyo, Japan) equipped with the Nikon NIS-Elements D at 40× magnification. Finally, adherent monocytes were counted using the ImageJ program (http://imagej.nih.gov/ij/, accessed on 2 November 2022). 

### 2.9. Statistical Analysis

Statistical analysis complies with international recommendations on experimental design and analysis in pharmacology [59,60,61]. All data are presented as means ± SD, and were analyzed using one/two-way ANOVA followed by Dunnett’s for multiple comparisons. GraphPad Prism 8.0 software (San Diego, CA, USA) was used for analysis. Differences among groups were considered significant when a value of *p* ≤ 0.05 was achieved.

## 3. Results

### 3.1. Effect of TIIA and CRY on SGBS Adipocytes Viability

Preliminary experiments were conducted to test the effects of TIIA and CRY on cell viability. Fully differentiated SGBS adipocytes were pre-treated with TIIA or CRY at the concentration range of 0.1–50 µM (based on literature studies [40,62]) for 1 h, and then stimulated with TNF-α 10 ng/mL for 18 h. After treatments, cell viability was determined by the MTT assay. 1–h pretreatment time was chosen as the most effective treatment time for both compounds in pilot time-course studies (6 h, 3 h, 1 h and 0 h pretreatment before TNF-α) evaluating their anti-inflammatory action against MCP-1 gene expression.

**Figure 2:**
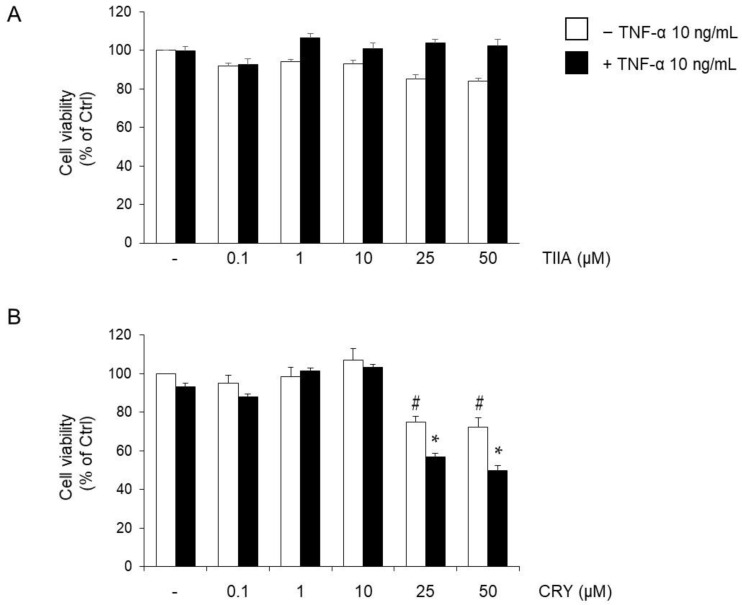
The effect of TIIA and CRY treatment on cell viability. SGBS adipocytes were treated with TIIA (**A**) or CRY (**B**) for 1 h at the concentrations indicated, and then either treated with 10 ng/mL TNF-α (black filled bars), or left untreated (open white bars) for 18 h. Cell viability was assessed by the MTT assay, and expressed as a percent of unstimulated control (Ctrl). Data are means ± SD (*n* = 3). # *p* < 0.05 versus Ctrl. * *p* < 0.05 versus TNF-α alone.

As shown in Figure 2A, TIIA at concentrations tested in the absence or presence of TNF-α did not significantly affect cell viability. Similar results were seen morphologically or observed at the protein level. CRY reduced adipocyte viability at concentrations ≥25 µM, in the absence or presence of TNF-α (Figure 2B). Thus, we used 10 μM as the concentration of TIIA and CRY for further experiments.

### 3.2. TIIA and CRY Decrease the Release of Cyto/Chemokines after TNF−α Stimulation

**Figure 3:**
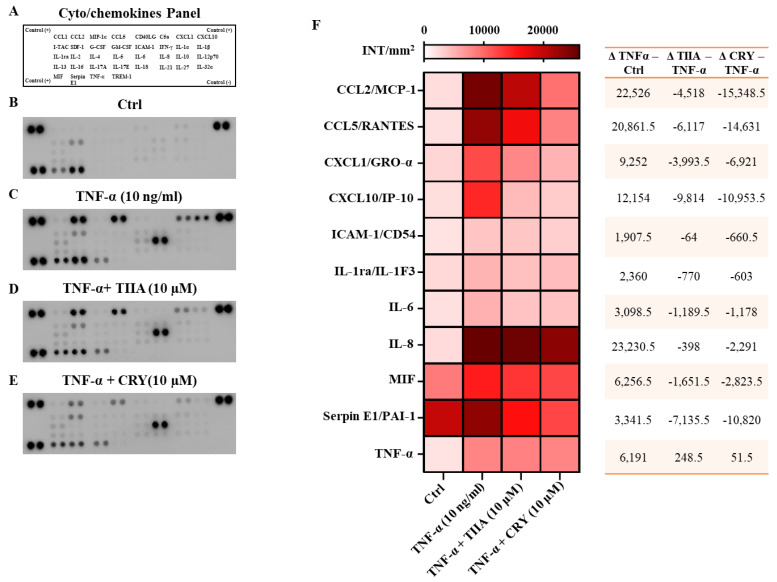
Cell supernatants were assayed using a proteome profiler cytokine array (**A**) for Ctrl (**B**), TNF−α (10 ng/mL) (**C**), TNF−α + TIIA 10 μM (**D**) and TNF−α + CRY 10 μM (**E**) groups. Densitometric analysis is presented as a heatmap, expressed as INT(intensity)/mm^2^ (**F**). On the right panel of the heatmap is reported a table list with Δ values (Δ TNF−α−Ctrl; Δ TIIA−TNF-α; Δ CRY−TNF−α) of all pro-inflammatory cyto/chemokines. Data are presented as means ± SD of positive spots of three separate independent experiments run each with *n* = 3 per group pooled (**F**). ElisaSpot assay statistical analysis (reported in the text) was performed by using two-way ANOVA followed by Dunnett’s for multiple comparisons.

The effect of TIIA and CRY on TNF−α-induced secretion of a panel of pro-inflammatory cyto/chemokine was evaluated (panel reported in Figure 3A). Among the anti-/pro-inflammatory cyto/chemokines tested, cell supernatants obtained from TNF−α (10 ng/mL) group (Figure 3C) displayed a large increase of pro-inflammatory cyto/chemokines compared to Ctrl (Figure 3B). Interestingly, when comparing cell supernatants from TIIA- and CRY-treated groups (both at 10 μM) with TNF−α (alone), we observed a significant decrease in several mediators (Figure 3D,E). Densitometric analysis, presented as a heatmap, revealed a significant reduction of the following TNF−α-stimulated pro-inflammatory factors: MCP-1/CCL2 (*p* ≤ 0.0001), CCL5/RANTES (*p* ≤ 0.0001), (CXCL1/GRO-α (*p* ≤ 0.0001), CXCL10/IP-10 (*p* ≤ 0.0001), IL-6 (*p* ≤ 0.05), IL-8/CXCL8 (*p* ≤ 0.0001, only for CRY), macrophage migration inhibitory factor (MIF) (*p* ≤ 0.001 and *p* ≤ 0.0001 for TIIA and CRY, respectively) and plasminogen activator inhibitor 1 (PAI-1/Serpin E1) (*p* ≤ 0.0001) (Figure 3F).

### 3.3. TIIA and CRY Attenuate Monocyte Adhesion to Inflamed Adipocytes

**Figure 4:**
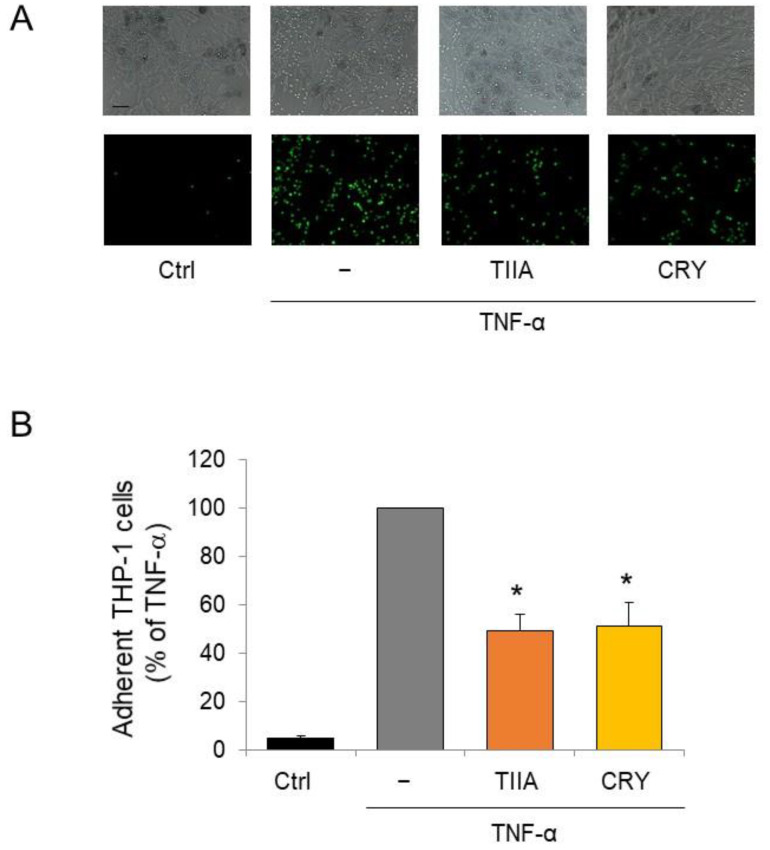
Modulation by TIIA and CRY of THP−1 monocytes adhesion to inflamed adipocytes. Fluorescently labeled THP−1 (10^6^ cells/mL) were added to SGBS monolayers pretreated with 10 μM TIIA or CRY before stimulation with 10 ng/mL TNF−α for 18 h. After 1 h, non-adhering cells were removed and (**A**) images of SGBS and adherent calcein-labeled THP−1 cells were visualized and captured with a phase contrast microscope (upper panels) and a fluorescent microscope (lower panels). (**B**) The number of adherent THP−1 cells is expressed as a percent of TNF-α. Scale bar = 50 µm. * *p* < 0.05 versus TNF-α alone.

As a functional counterpart of the modulation of adipocyte secretion profiles by TIIA and CRY, we assessed the effect of both compounds on the adhesion of monocytes to inflamed adipocytes. As shown in Figure 4, TNF-α stimulated the adhesion of THP-1 monocytes on adipocytes as a consequence of the induced secretion of cytokines and chemokines. Contrarily, TIIA and CRY treatment reduced the adhesion of THP-1 monocytes on TNF-α-stimulated adipocytes, in line with the regulation of adipokines by TIIA and CRY toward an anti-inflammatory profile.

### 3.4. TIIA and CRY attenuate TNF-α–Mediated Inflammatory Gene Expression in Human Adipocytes

**Figure 5:**
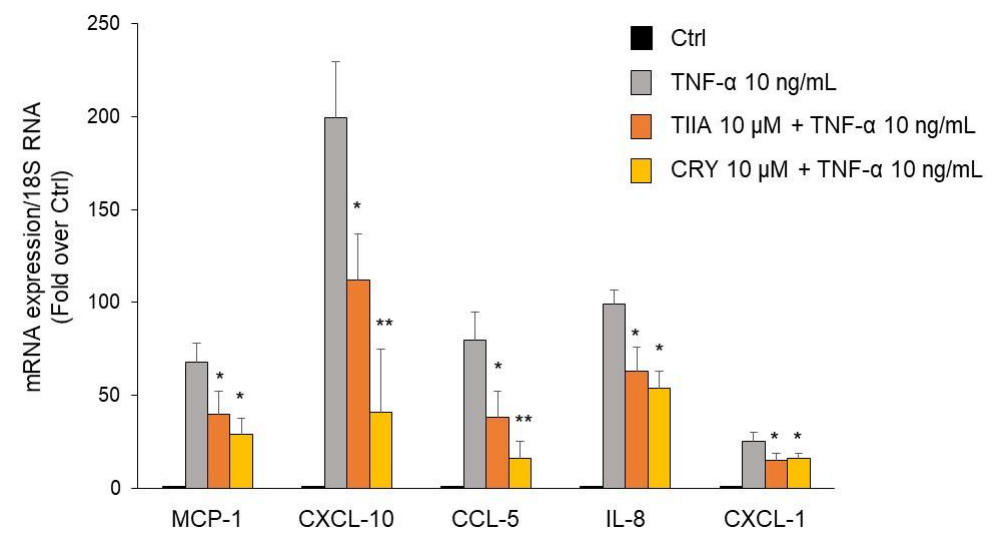
Modulation by TIIA and CRY of mRNA expression levels of genes associated with adipocyte inflammation. SGBS adipocytes were pretreated with 10 µM TIIA or CRY (1 h) at the concentrations indicated, and then treated with 10 ng/mL TNF-α for 18 h. Total RNA was extracted from cells, and mRNA levels of the indicated genes were measured by qPCR using specific primers and probes and normalized to 18S RNA. Data (means ± SD, *n* = 3) are expressed as fold induction over unstimulated control (Ctrl). * *p* < 0.05 versus TNF-α alone. ** *p* < 0.01 versus TNF-α alone.

To evaluate the modulation of secretory proteins by TIIA and CRY at the transcriptional level, mRNA expression levels of MCP-1, CXCL-10, CXCL-1, CCL-5, and IL-8 were determined by qPCR. As shown in Figure 5, both compounds were able to significantly downregulate the TNF-α-stimulated increase in the mRNA expression of the tested chemokines and cytokines, with a more potent inhibitory effect of CRY compared with TIIA, in accordance with the modulation of corresponding protein release.

### 3.5. TIIA and CRY Modulate TNF-α-Induced Inflammation-Linked miRNAs Expression in Adipocytes

**Figure 6:**
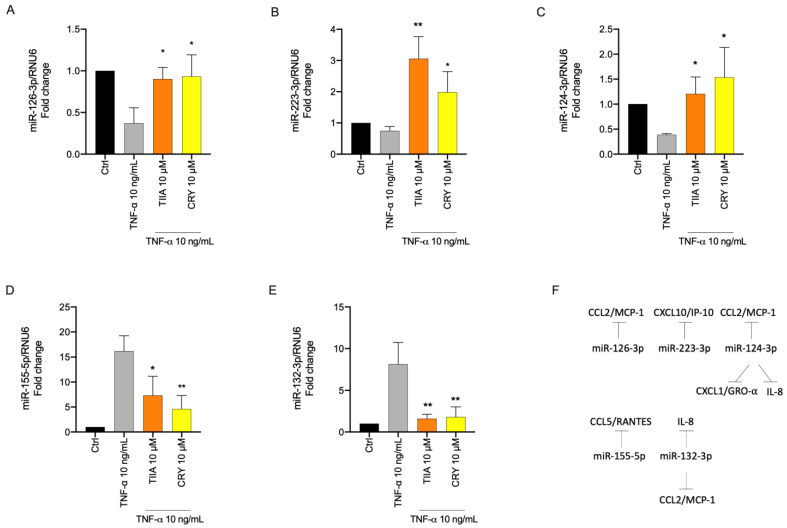
Modulation by TIIA and CRY of miRNA expression levels associated with inflammation. SGBS adipocytes were pretreated with 10 µM TIIA or CRY (1 h) and then treated with 10 ng/mL TNF−α for 18 h. Total miRNAs were extracted from cells, and miRNA levels were measured by qPCR and normalized to RNU6. (**A**) miR−126−3p, (**B**) miR−223−p, (**C**) miR−124−3p, (**D**) miR−155−5p, (**E**) miR−132−3p. (**F**) Schematic representation of the miRNA-mRNA targeting, arrow: direct inhibition, dotted arrow: indirect inhibition. Data (means ± SD, *n* = 3) are expressed as fold change (2–ΔΔCt) versus unstimulated control (Ctrl). * *p* < 0.05 versus TNF−α alone. ** *p* < 0.01 versus TNF-α alone.

To evaluate the modulatory effect of the tested diterpenoids on miRNAs expression related to inflammation, SGBS adipocytes were pre-incubated with TIIA or CRY for 1 h and then stimulated with TNF-α for 18 h. Levels of miRNAs extracted from adipocytes were analyzed by qPCR. Under inflammatory conditions induced by TNF-α, we observed a decrease of miRNA known as anti-inflammatory i.e., miR-126-3p, miR-223-3p, and miR-124-3p (Figure 6A–C), and an increased expression of miRNAs known as pro-inflammatory such as miR-155-5p and miR-132-3p, compared to levels found in untreated cells (Figure 6D,E). Pre-treatment with TIIA or CRY significantly prevented the TNF-α-induced decreased expression of miR-126-3p, miR-223-3p, and miR-124-3p (Figure 6A–C). In parallel, treatment with TIIA or CRY significantly counteracted the higher expression of miR-132-3p and miR-155-5p observed under TNF-α alone (Figure 6D,E).

### 3.6. Biological Processes Associated to miRNAs Modulated by TIIA and CRY in Inflamed Adipocytes

**Figure 7:**
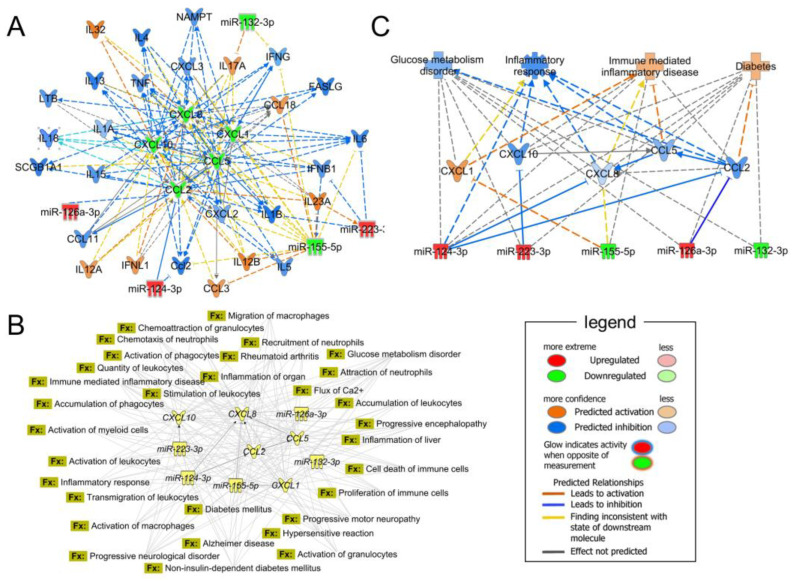
Bioinformatics survey of miRNA-mRNA modulated by TIIA and CRY. (**A**) Assessment of miRNA and genes regulation by a prediction network encompassing pro-inflammatory cytokines connected with at least two target miRNA/mRNA and bioinformatically predicted to be activated or inhibited by their modulation. (**B**) Functional association network generated according to the relationship of significant functions and the target miRNAs/genes. Significant molecular functions with less than 5 connections were not included. (**C**) Hierarchical network predicting the effect of miRNAs up/down-regulation on gene expression and the downstream effects on the top *Disease and Function* annotations.

A bioinformatic analysis of upregulated miR-124-3p, miR-126-3p, miR-223-3p and downregulated miR-155-5p, miR-132-3p, and the mRNAs for CCL2, CCL5, CXCL1, CXCL8, and CXCL10 were conducted. The miRNA–mRNA network showed that they participated in the regulation of immune response and inflammation mediated by other cytokines that are at least double-connected and predicted to be activated or inhibited by the modulation of miRNA/mRNA targets (Figure 7A). This approach allowed us to identify a set of relevant pro-inflammatory cytokines acting in connection with our miRNA/mRNA. Besides representing potential future targets, cytokines such as IL-1, IFN-γ, TNF-α, or IL-6 appear to be themselves influenced by TIIA and CRY treatment.

Ingenuity knowledge base, a repository of biological interactions and functional annotations created from multiple individually modeled cellular relationships, was used to predict the possible functions significantly involved in the miRNA/mRNA modulation (Figure 7B). Downstream analysis, which scrutinized the main functions involving at least 5 out of 10 nodes with a *p* value < 0.01, revealed the presence of five macro-categories of interest, including *Metabolic disorders*, *Neurological diseases*, *Inflammatory disease and response*, *Hematological system development and function* and *Cell signaling.*

Furthermore, we hierarchically connected miRNA, genes and the main *Disease and Function* with higher values of predicted activation/inhibition and lower *p*-value (Figure 7C). Also, in this case, annotations related to metabolic disorders and inflammation appeared to be the most involved in the modulation of our targets.

## 4. Discussion

In the present study, we investigated gene, miRNAs and protein expression profiles of prototypical obesity-associated dysfunction markers in inflamed human adipocytes treated with TIIA and CRY, the two most important lipophilic diterpenoid compounds of *Salvia milthorrhiza* Bunge. The results showed that TIIA and CRY prevented TNF-α-induced inflammatory response in adipocytes, by counter-regulating the pattern of secreted cytokines/chemokines associated with adipocyte inflammation (CCL2/MCP-1, CXCL10/IP-10, CCL5/RANTES, CXCL1/GRO-α, IL-6, IL-8, MIF and PAI-1/Serpin E1) via the modulation of gene expression (as demonstrated for CCL2/MCP-1, CXCL10/IP-10, CCL5/RANTES, CXCL1/GRO-α, and IL-8), as well as related miRNA expression (miR-126-3p, miR-223-3p, miR-124-3p, miR-155-5p, and miR-132-3p). This is the first demonstration of a beneficial effect of TIIA and CRY on adipocyte dysfunction associated with obesity development and complications, thus opening new perspectives in pharmacological research for the prevention and/or treatment of metabolic diseases.

The altered pattern of adipokine production by hypertrophic adipocytes is a typical marker and a pathophysiological feature of visceral obesity [63]. Adipokines may exert both local and systemic roles: at the adipose tissue level, they regulate adipogenesis, insulin sensitivity, adipocyte metabolism and function, and immune cell recruitment and activation [63]. At the systemic level, they regulate appetite/satiety, energy metabolism, body fat distribution, insulin secretion and action, blood pressure, vascular function, and hemostasis, thus implying more widespread endocrine effects of adipose tissue inflammation. Obesity is associated with the upregulation of the expression and secretion of pro-inflammatory, diabetogenic and pro-atherogenic factors that therefore represent a pathogenic link–and a targetable component–between obesity and related metabolic and cardiovascular complications [63,64].

TNF-α is a prototypical pro-inflammatory adipocytokine whose expression, mostly by adipocytes as well as adipose tissue macrophages, increases during the development of obesity [46,49], and contributes to adipocyte dysfunction by inhibiting adipogenesis and insulin signaling and inducing lipolysis and inflammation [65,66]. Accordingly, we observed that adipocytes exposure to TNF-α led to increased levels of secreted adipokines i.e., CCL2/MCP-1, CXCL10/IP-10, CCL5/RANTES, CXCL1/GRO-α, IL-6, IL-8, MIF and PAI-1/Serpin E1, and corresponding mRNAs as surveyed for a subset of adipokines i.e., CCL2/MCP-1, CXCL10/IP-10, CCL5/RANTES, CXCL1/GRO-α, and IL-8. This was functionally accompanied, as occurs during obesity development, by the adhesion of THP-1 monocytes on inflamed adipocytes as a consequence of the induced adipocyte secretion of cytokines and chemokines.

Chemokines (or chemoattractant cytokines) play a pivotal role in the recruitment and inflammatory activation of immune cells at sites of chronic inflammation, including the expanding adipose tissue in obesity [67]. The contributory role of the chemokine CCL2/MCP-1 in macrophage recruitment in obesity in metabolic tissues and consequent insulin resistance has been shown early in animal models [11,12,68,69].

However, the genetic deficiency or inactivation of CCL2/MCP-1 or its receptor (CCR2) did not entirely restrain macrophage infiltration in obese adipose tissue, suggesting other chemokines may be involved [12]. In support of this, CCL5/RANTES has also been found to be overexpressed in obese adipose tissue [70,71], and implicated in macrophage accumulation and survival, inflammation and insulin resistance in the visceral adipose tissue during obesity [72,73]. Although CCL5 is thought to be mainly expressed by the stroma-vascular fraction of adipose tissue in obesity [74] and its deficiency led to a compensatory increase in T cell recruitment [75], adipocytes may also be an important source of CCL5 as demonstrated in previous findings [70], as well as in the present study. CXCL10/IP-10 is another potent chemotactic factor with higher expression in obese adipose tissue [70,76] and circulating levels significantly associated with visceral adipose tissue expansion, insulin resistance and metabolic syndrome [77]. Moreover, CXCL1/GRO-α and IL-8 also exhibited higher expression in the adipose tissue of obese subjects [70] as well as in the circulation of obese mice and humans in association with insulin resistance and type 2 diabetes [78,79]. Interestingly, several of these chemokines were shown to promote the recruitment of other immune cells besides macrophages, such as neutrophils (as for CXCL1 and IL-8) [80] and Th1 (pro-inflammatory) cells (as for CCL5 and CXCL-10/IP-10) [81], that also infiltrate and derange adipose tissue in response to obesity [81,82]. MIF is a pleiotropic cytokine exerting pro-inflammatory and dysmetabolic functions and exhibiting higher adipose tissue and plasma levels in obesity, insulin resistance, and type 2 diabetes [83,84]. PAI-1 is an adipokine that is highly expressed in adipose tissue in obesity, insulin resistance, and type 2 diabetes, having pro-inflammatory and pro-thrombotic functions that may play a role in impaired fibrinolysis associated with obesity [85,86]. IL-6 is another cytokine that can be produced by adipocytes and is crucially implicated in the pathogenesis of obesity and insulin resistance [87,88,89]. Circulating levels of IL-6 positively correlate with obesity in humans [88] and predict the risk of insulin resistance and type 2 diabetes [87]. Notably, IL-6 produced by visceral adipose depots is drained directly into the portal circulation and contributes to obesity-associated production of C reactive protein (CRP), a clinical index of cardiovascular risk [90].

We observed that TIIA and CRY were able to significantly downregulate the TNF-α-induced production of these cytokines and chemokines in adipocytes, along with a concordant inhibition of monocyte recruitment. Given the role of these adipokines in obesity and its adverse sequelae, we can hypothesize that the observed inhibitory effects by TIIA and CRY may attenuate the adipose tissue and systemic chronic inflammatory tone associated with obesity and the metabolic and vascular consequences [91].

Previous findings support the multifaceted pharmacological properties of the diterpenes TIIA and CRY. In preclinical studies, anti-cancer, cardioprotective, vasculoprotective, anti-platelet, hepatoprotective, neuroprotective, and antidiabetic effects have been attributed to both the active molecules of *S. milthorrhiza* [28,62,92,93]. Most of the benefits of both compounds are thought to derive from the ability to blunt inflammation as shown in several cell and animal models, for example by inhibiting: (1) the production of pro-inflammatory molecules, including some of those here investigated, adhesion molecules, pro-inflammatory or matrix remodeling enzymes; (2) the activation of signaling pathways such as mitogen-activated protein kinase (MAPK) and NLRP3 inflammasome, and of inflammatory transcription factors such as NF-κB and signal transducer and activator of transcription (STAT); and (3) by decreasing the overproduction of reactive oxygen species and/or inducing the cytoprotective antioxidant response via activation of Nrf2 [62,92,94].

Concerning metabolic diseases and especially adipocyte function, anti-obesity effects have been documented for TIIA and CRY. In animal models of obesity and NAFLD, TIIA attenuated weight gain, dyslipidemia, plasma levels of IL-6, TNF-α and IL-1β, and oxidative stress markers by inhibiting toll-like receptor (TLR)4/NF-κB signaling pathway and upregulating PPARγ [95]. Moreover, TIIA suppressed 3T3-L1 preadipocyte differentiation through antagonism of PPARγ transcriptional activities and decreased adipose mass, body weight, and improved glucose tolerance and blood lipid levels in high-fat diet-induced obese mice [96]. CRY promoted mitochondrial biogenesis and brown differentiation in mesenchymal stem cells [97]. This is in line with the in vivo [98] and in vitro [99] evidence of anti-obesity effect by CRY [98,99]. Beyond anti-adipogenic effects, insulin-sensitizing activity and anti-diabetic effects have been reported for TIIA [100,101,102], and CRY [103]. In accordance with these literature data, we here added novel evidence of potential anti-obesity effects through the modulation of the inflammatory response in adipocytes which is a pivotal process in the development of metabolic complications.

As a part of our investigation into the efficacy of TIIA and CRY on adipocyte inflammation, we evaluated their effects on epigenetic regulators of gene expression and specifically on miRNA, a small non-coding RNA molecule that functions to regulate gene expression by repressing translation or cleaving mRNAs [104,105]. There has been a growing interest in these molecules as potential disease biomarkers and therapeutic targets because a single miRNA can directly bind to several transcripts, affecting their expression and thereby simultaneously controlling a wide range of biological processes, such as adipogenesis, insulin signaling, adipokine expression, inflammation, and food intake [106,107,108,109].

The present study showed that TIIA and CRY can attenuate miRNA expression changes in human adipocytes induced by TNF-α. Indeed, TNF-α upregulated miR-155-5p and miR-132-3p and downregulated miR-126-3p, miR-223-3p and miR-124-3p. On the contrary, part of the anti-inflammatory effects of TIIA and CRY are likely to be explained by their ability to restore miRNAs expression in inflamed adipocytes. In detail, TIIA and CRY decreased the TNF-α-stimulated levels of CCL2/MCP-1 and CXCL-10/IP-10 mRNA and protein expression and, correspondingly, increased the levels of their inhibitory miRNAs, i.e., miR-126-3p [110], miR-223-3p [111] and miR-124-3p [112]. Furthermore, TIIA and CRY significantly decreased TNF-α-induced expression of IL-8 and CXCL-1/GRO-α and increased their experimentally validated inhibitory miR-124-3p [112]. TIIA and CRY also inhibited TNF-α-stimulated expression of miR-132-3p, indirectly reducing the expression of CCL2/MCP-1 and IL-8 [113]. Finally, both tested compounds attenuated TNF-α-stimulated expression of miR-155-5p, which may indirectly decrease CCL-5/RANTES expression in an NF-κB-dependent manner [114]. In line with our data, miR-155 is increased in obese adipose tissue [115] and is upregulated by TNF-α in adipocytes via NF-κB activation [116]. Interestingly, miR-155-5p is considered a master regulator of inflammation [117] and is involved in the pathogenesis of obesity and insulin resistance [118,119]. A similar modulatory effect by TIIA on miR-155-5p was observed in RAW264.7 macrophages incubated with LPS [36,37]. miR-155 inhibition may translate into reduced pro-inflammatory activation being miR-155 involved in pro-inflammatory M1 polarization of macrophages [120]. On the other hand, miR-223, which was upregulated by TIIA and CRY, was shown to protect against adipose tissue inflammation and systemic insulin resistance [121].

The regulation of miRNAs by the two diterpenes has been previously shown in a few studies concerning different pathophysiological settings, such as cardiovascular diseases [122,123,124] or cancer [125]. Our data, therefore, strengthen and expand evidence on potential molecular mechanisms of the anti-inflammatory action of TIIA and CRY, and indicate that TIIA and CRY exert their anti-inflammatory activity in adipocytes also by controlling miRNAs expression towards an anti-inflammatory profile that may improve adipocyte dysfunction. Notably, miRNAs can be packaged into exosomes that can regulate gene expression in a paracrine fashion and distant organs [118,126]. miRNAs, besides adipokines, may represent a significant and novel targetable way for the adipose tissue to cross-talk with other cells/tissues and contribute to local and systemic chronic inflammation in obesity.

Aiming at providing further functional insight into the effects of TIIA and CRY in adipocytes, the integrative bioinformatic analysis of the mRNA-miRNA networks revealed that other inflammation-related cytokines and chemokines were predicted to be affected by the diterpenes, thus potentially expanding the influence of these compounds on the networks of immune/inflammatory mediators. Moreover, several enriched functions, such as immune cell chemoattraction, migration and activation, or dysmetabolism, relevant to inflammatory and cardiometabolic diseases were identified among the most significantly modulated by TIIA and CRY. This analysis may allow a better understanding of the molecular mechanisms of action of TIIA and CRY [92] and provide avenues for identifying potential target genes and pathways for future studies.

In vivo studies conducted in mice reported the anti-inflammatory and anti-oxidant propriety of TIIA and CRY in a dosage range between 10–90 mg/kg [35,38,39,40,95,98,102]. Therefore, we estimated a human equivalent dose (HED) of 0.67–6.09 mg/kg and an estimated human dose of 47.4–426.3 mg/die in an adult of 70 kg [127]. These speculations could serve as the starting point for future studies aimed at supporting the clinical use of TIIA and CRY.

## 5. Conclusions

Our study demonstrates that TIIA and CRY ameliorate adipocyte dysfunction associated with obesity via the modulation of miRNA, gene, and protein expression toward an anti-inflammatory phenotype. Pending experimental studies to further extrapolate these effects in patients, these data recognize TIIA and CRY as natural compounds with attractive biological and pharmacological properties for the development of novel drugs/supplements for clinical use.

## Figures and Tables

**Table 1:** Primer sequences used for qPCR analysis.

Gene	Forward Primer	Reverse Primer
CCL2/MCP-1	5′-CCCCAGTCACCTGCTGTTAT-3′	5′-TCCTGAACCCACTTCTGCTT-3′
CXCL1/GRO-α	5′-CCCCAAGAACATCCAAAGTG-3′	5′-TGGATTTGTCACTGTTCAGCA-3′
CXCL10/IP-10	5′-CAAGGATGGACCACACAGAG-3′	5′-GCAGGGTCAGAACATCCACT-3′
CCL5/RANTES	5′-CGCTGTCATCCTCATTGCTA-3′	5′-GAGCACTTGCCACTGGTGTA-3′
IL-8	5′-GTGCAGTTTTGCCAAGGAGT-3′	5′-CTCTGCACCCAGTTTTCCTT-3′
18S	5′-AAACGGCTACCACATCCAAG-3′	5′-CCTCCAATGGATCCTCGTTA-3′

MCP-1/CCL-2: monocyte chemoattractant protein-1/chemokine (C-C motif) ligand 2; CXCL-1/GRO- α; chemokine (C-X-C motif) ligand 1/growth regulated protein α; CXCL-10/IP-10: chemokine (C-X-C motif) ligand 10/interferon γ-induced protein-10; CCL-5/RANTES: chemokine (C-C motif) ligand-5/regulated on activation, normal T cell expressed and secreted; IL-8: interleukin-8.

**Table 2:** Mature miRNAs sequences and miRBase Accession number.

miRNA Name	Mature miRNA Sequences	miRBase Accession
hsa-miR-126-3p	5′-UCGUACCGUGAGUAAUAAUGCG-3′	MIMAT0000445
hsa-miR-223-3p	5′-UGUCAGUUUGUCAAAUACCCCA-3′	MIMAT0000280
hsa-miR-124-3p	5′-UAAGGCACGCGGUGAAUGCC-3′	MIMAT0000422
hsa-miR-155-5p	5′-UUAAUGCUAAUCGUGAUAGGGGU-3′	MIMAT0000646
hsa-miR-132-3p	5′-UAACAGUCUACAGCCAUGGUCG-3	MIMAT0000426

## Data Availability

The data presented in this study are available on request from the corresponding author.
